# Identifying neurocognitive disorder using vector representation of free conversation

**DOI:** 10.1038/s41598-022-16204-4

**Published:** 2022-08-03

**Authors:** Toshiro Horigome, Kimihiro Hino, Hiroyoshi Toyoshiba, Norihisa Shindo, Kei Funaki, Yoko Eguchi, Momoko Kitazawa, Takanori Fujita, Masaru Mimura, Taishiro Kishimoto

**Affiliations:** 1 Department of Neuropsychiatry, Keio University School of Medicine, Tokyo, Japan; 2 Lifescience AI Business Division, Research Development Department, FRONTEO Inc, Tokyo, Japan; 3 Department of Health Policy and Management, Keio University School of Medicine, Tokyo, Japan; 4 Hills Joint Research Laboratory for Future Preventive Medicine and Wellness, Keio University School of Medicine, 7th Floor, Roppongi Hills North Tower, 6-2-31 Roppongi, Minato-ku, Tokyo, 106-0032 Japan; 5 Psychiatry at Donald and Barbara Zucker School of Medicine at Hofstra/Northwell, New York, USA

**Keywords:** Biomarkers, Health care, Medical research, Signs and symptoms

## Abstract

In recent years, studies on the use of natural language processing (NLP) approaches to identify dementia have been reported. Most of these studies used picture description tasks or other similar tasks to encourage spontaneous speech, but the use of free conversation without requiring a task might be easier to perform in a clinical setting. Moreover, free conversation is unlikely to induce a learning effect. Therefore, the purpose of this study was to develop a machine learning model to discriminate subjects with and without dementia by extracting features from unstructured free conversation data using NLP. We recruited patients who visited a specialized outpatient clinic for dementia and healthy volunteers. Participants’ conversation was transcribed and the text data was decomposed from natural sentences into morphemes by performing a morphological analysis using NLP, and then converted into real-valued vectors that were used as features for machine learning. A total of 432 datasets were used, and the resulting machine learning model classified the data for dementia and non-dementia subjects with an accuracy of 0.900, sensitivity of 0.881, and a specificity of 0.916. Using sentence vector information, it was possible to develop a machine-learning algorithm capable of discriminating dementia from non-dementia subjects with a high accuracy based on free conversation.

## Introduction

The number of people with dementia is increasing around the world, and so is the financial burden^1^. Early intervention for dementia has many benefits, such as delaying progression^2–4^, maintaining the patient's activities of daily living and quality of life^3,5^, and reducing care costs^6,7^. However, the diagnosis of dementia is not easy, especially in the early stages^8,9^, and simple, non-invasive methods of early detection are needed. Recent studies have reported that Alzheimer's disease (AD) and non-dementia subjects can be distinguished with a high accuracy by extracting features from spontaneous speech using natural language processing (NLP) and machine learning^10–18^. For example, Orimaye et al. used a picture description task to record the spontaneous speech of 99 AD patients and 99 cognitively healthy control (CHC) individuals and attempted to discriminate these groups by applying machine learning using syntactic and lexical features^11^. As a result, they reported a high discrimination accuracy of Area Under the Curve (AUC) 0.93. Similar to their study, which used NLP and machine learning to discriminate dementia from CHC, the mainstream approach is to use a picture description task to encourage spontaneous speech. However, we were interested to see whether linguistic changes associated with cognitive decline can also be observed during non-task-based free conversation. It has been pointed out that the differences in speech between AD patients and CHC observed under specific tasks such as picture description are not necessarily also observed in free conversation^19^. Nevertheless, most studies have been validated with the same audio dataset, DementiaBank, obtained from subjects who were asked to describe the Cookie Theft Picture. In the past, there have been few studies in which spontaneous speech was encouraged in interview-style conversations and the recorded data was used for NLP^16–18^. Guinn et al. attempted to discriminate 31 AD and 57 controls from their interview-style conversations by machine learning using features such as lexicosyntactics features and the rate of speech^16^. The maximum accuracy was 0.795 (sensitivity 0.419, specificity 1.0). Jarrold et al. attempted to discriminate 9 AD and 9 controls by machine learning using lexical and acoustic features as features in the recorded semi-structured interview data^17^. The maximum discrimination accuracy between AD and control was 0.88 (sensitivity 0.83 specificity 0.90). Mirheidari et al. recorded the conversations of 15 patients with neurodegenerative disorders and 15 patients with Functional Memory Disorder who visited a memory clinic while being examined by a neurologist^18^. Then they attempted to discriminate between the two groups by machine learning, using lexical features, acoustic features, and subjects' verbal responses to specific questions (e.g., who is most concerned about their symptoms?) and non-verbal information such as 'patient turns to other' as features. The results showed a maximum accuracy of 0.97, but the lexical features had the smallest contribution to the prediction. All of these studies were conducted on a small number of cases. On the other hand, studies using the DementiaBank dataset were limited to subjects with AD, however, it is necessary to evaluate cognitive function regardless of the cause of dementia in order to apply them to real-world clinical practice. Therefore, the purpose of this study was to create a machine learning model to discriminate dementia, including non-AD, and non-dementia subjects by applying NLP to unstructured free conversation data.

## Results

### Datasets

**Figure 1:**
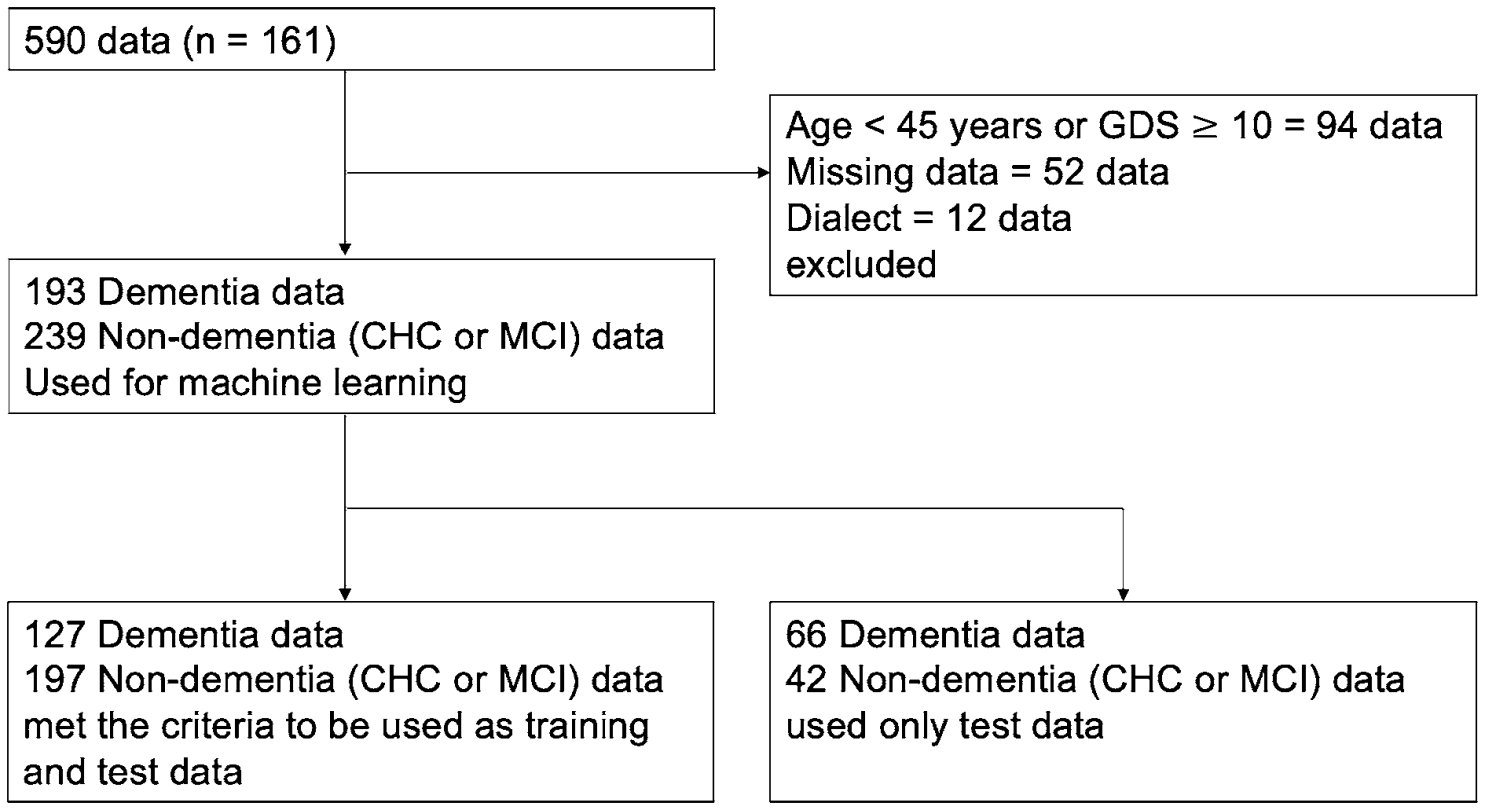
Number of datasets used for training and testing. GDS: Geriatric Depression Scale. CHC: cognitively healthy control. MCI: mild cognitive impairment.

A total of 590 datasets (n = 161) were collected as a total study sample, including dementia, mild cognitive impairment (MCI), and CHC. For this study, 432 (n = 135) of the 590 datasets were analyzed after excluding datasets collected from participants who were under 45 years old, data with a GDS score of 10 or higher, data with missing data, and data in which subjects spoke in a strong dialect (Fig. 1). The final analysis dataset consisted of 162 datasets from men (n = 58; mean age, 74.4 ± 9.4 years) and 270 datasets from women (n = 77; mean age, 74.8 ± 11.7 years), with 193 dementia datasets (n = 58; mean age, 79.0 ± 8.9 years) and 239 non-dementia datasets (n = 83; mean age, 71.1 ± 11 0.1 years). Of these, 127 dementia datasets (n = 47; mean age, 79.2 ± 9.8 years) and 197 non-dementia datasets (n = 78; mean age, 70.5 ± 11.6 years) met the criteria for training data. See Table 1 for the participants’ demographic characteristics.

**Table 1:** Demographic data.

	Total	Dementia	Non-dementia	Dementia data that meets the criteria for training	Non-dementia data that meets the criteria for training
Dataset (% female)	432 (62.5)	193 (76.7)	239 (51.0)	127 (85.8)	197 (54.8)
n (% female)	135 (57.0)	58 (70.7)	83 (47.0)	47 (78.7)	78 (47.4)
Age (mean ± SD), in years	74.6 ± 10.9	79.0 ± 8.9	71.1 ± 11.1	79.2 ± 9.8	70.5 ± 11.6
MMSE (mean ± SD)	23.2 ± 7.0	16.4 ± 4.8	28.6 ± 1.8	14.9 ± 4.8	28.7 ± 1.6
CDR (mean ± SD)	0.6 ± 0.8	1.3 ± 0.7	0.1 ± 0.2	1.6 ± 0.5	0.1 ± 0.2
LM II (mean ± SD)	6.8 ± 7.1	0.5 ± 1.2	11.8 ± 5.8	0.3 ± 0.8	12.5 ± 5.9
Letters (mean ± SD)	2161.6 ± 880.8	1755.7 ± 876.0	2489.3 ± 737.8	1622.6 ± 857.9	2508.4 ± 775.9

Non-dementia includes cognitively healthy controls and participants with mild cognitive impairment. Criteria for training: criteria for datasets obtained from neuropsychological tests to be used as training data for machine learning.

*MMSE* mini-mental state examination, *CDR* clinical dementia rating, *LM II* logical memory delayed recall of Wechsler Memory Scale-Revised, Letters: Number of letters uttered by the subject in the recorded free conversation.

### Prediction accuracy

**Figure 2:**
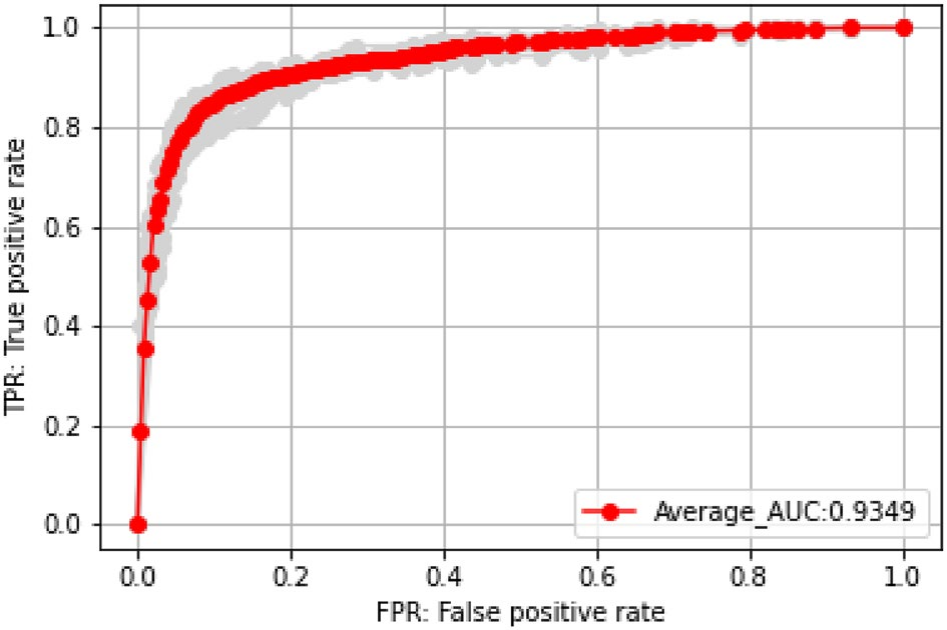
Receiver Operating Characteristic (ROC) curve of machine learning model.

A threshold of 1, which was the threshold with the highest prediction accuracy, was adopted for the number of votes for the prediction model. The results for discriminating between dementia and non-dementia using all the datasets showed an accuracy of 0.900, a sensitivity of 0.881, and a specificity of 0.916 (Table 2). Average AUC was 0.935 (Fig. 2). There was no statistically significant difference between the prediction accuracy for the data that met the criteria for use as training data and that of the overall data (χ^2^ = 0.402, *p* = 0.526). There was no statistically significant difference in the prediction accuracy according to sex (χ^2^ = 0.015, *p* = 0.901). There was no statistically significant difference in the prediction accuracy between those 75 years of age and older and those less than 75 years (χ^2^ = 2.902, *p* = 0.088).

**Table 2:** Discrimination results between dementia and non-dementia groups using machine learning.

	Data	Incorrect	Accuracy	Sensitivity	Specificity	χ^2^	*p* value
All data	432	43	0.900	0.881	0.916	0.402	0.526
Data that meets the criteria for training	324	27	0.917	0.929	0.909	–	–
Male	162	17	0.895	0.733	0.957	0.015	0.901
Female	270	26	0.904	0.926	0.877	–	–
Age < 75 years	178	12	0.933	0.881	0.949	2.902	0.088
Age ≥ 75 years	254	31	0.878	0.881	0.874	–	–

### Number of letters and prediction accuracy

**Figure 3:**
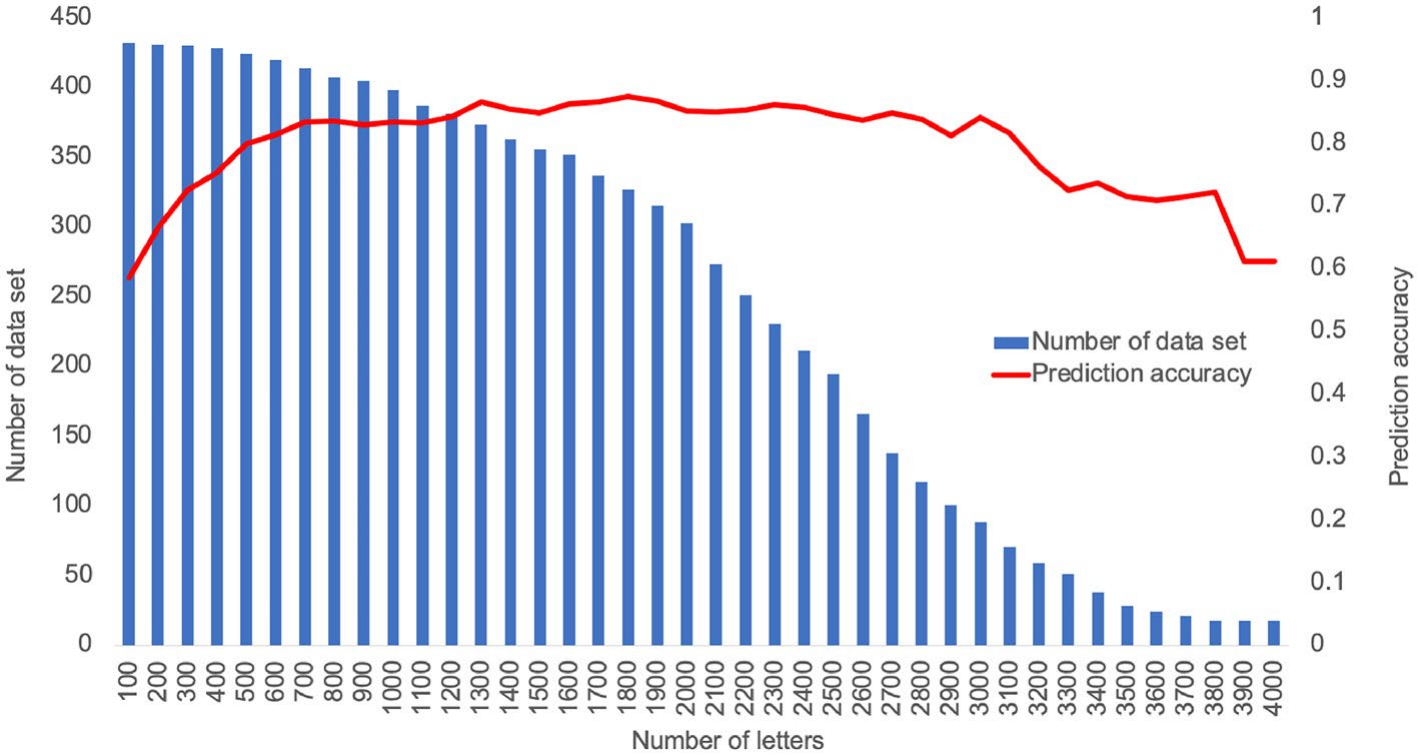
Relationship between number of letters and prediction accuracy of machine learning model.

The relationship between the number of letters in the text and the prediction accuracy is shown in Fig. 3. The prediction accuracy exceeded 0.8 at 600 letters and appeared to reach a plateau of 0.866 at 1300 letters. The peak was 0.875 at 1800 letters.

### Verification of vectorization and machine learning algorithms

The accuracy based on different numbers of voting for Deep Neural Network (DNN) and XGBoost using our original vector as well as DNN using Term Frequency–Inverse Document Frequency (TF-IDF) vector and Bidirectional Encoder Representations from Transformers (BERT) vector are shown in Table 3, with one vote using our original vector and DNN showed the highest accuracy. Including the above, the accuracy, sensitivity and specificity using other document embeddings and machine learnings are shown in Table 4. It was found that the DNN for the data vectorized with the original algorithm had the highest prediction accuracy.

**Table 3:** Prediction accuracy by voting of XGBoost and DNN model.

Voting	Accuracy			
	DNN model for original algorithm vector	XGBoost model for original algorithm vector	DNN model for TF-IDF vector	DNN model for BERT vector
0	0.447	0.447	0.447	0.447
1	0.900	0.785	0.819	0.794
2	0.889	0.836	0.824	0.829
3	0.875	0.833	0.810	0.836
4	0.870	0.836	0.799	0.831
5	0.859	0.831	0.785	0.847
6	0.856	0.801	0.785	0.840
7	0.847	0.780	0.768	0.822
8	0.845	0.752	0.752	0.822
9	0.836	0.727	0.741	0.808
10	0.815	0.674	0.706	0.778

*DNN* deep neural network, *TF-IDF* term frequency–inverse document frequency, *BERT* bidirectional encoder representations from transformers.

**Table 4:** Results using other Document Embedding and machine learning models.

Document Embedding	Model	Accuracy	Sensitivity	Specificity
Original algorithm	DNN	0.900	0.881	0.916
	GNB	0.817	0.674	0.933
	LR	0.831	0.674	0.958
	SVC	0.863	0.756	0.95
	XGboost	0.829	0.731	0.908
TF-IDF	DNN	0.824	0.798	0.845
	GNB	0.78	0.617	0.912
	LR	0.752	0.482	0.971
	SVC	0.785	0.565	0.962
	XGboost	0.785	0.653	0.891
BERT	DNN	0.847	0.762	0.916
	GNB	0.833	0.762	0.891
	LR	0.447	1	0
	SVC	0.845	0.710	0.95
	XGboost	0.826	0.731	0.904

*TF-IDF* term frequency–inverse document frequency, *BERT* bidirectional encoder representations from transformers, *DNN* deep neural network, *GNB* Gaussian Naive Bayes, *LR* logistic regression, *SVC* support vector machine classifier.

## Discussion

In this study, we used a total of 432 datasets to build a machine learning model to detect cognitive decline corresponding to dementia using text data transcribed from unstructured free conversation audio data. The final machine learning model classified dementia and non-dementia data with an AUC 0.93, accuracy of 0.900, a sensitivity of 0.881, and a specificity of 0.916 based on the largest amount of data ever reported in a study aimed at detecting dementia using NLP and machine learning based only on free conversation.

Of the studies using the DementiaBank dataset, Alkenani et al. reported an accuracy of 0.95 and an AUC of 0.98 for using only linguistic features^20^. They use lexicosyntactics and character n-gram spaces as features and combine the predictions of multiple classifiers in heterogeneous ensemble methods. We considered that accuracy could be improved by using a corpus of spoken language rather than a corpus of written language. On the other hand, since there is the absence of available Japanese corpus of spoken language, we decided to use a method that does not require a large training data set. In machine learning, we also decided to attempt to use DNN. It is important to note that, although our model did not compete with their AUC, our free conversation NLP, which does not use a task, was still accurate enough for screening.

The most widely used screening for dementia in real-world clinical practice is the MMSE. The sensitivity and specificity of the MMSE are reported to be 0.81 and 0.89, respectively^21^, and the results of this study are similar or even better, with a sufficient screening ability. In terms of the relationship between the number of letters and the prediction accuracy, we obtained an accuracy of 0.8 at 600 letters. Since daily conversation in Japanese is considered to be between 360 and 420 letters per minute, this means that we should be able to obtain 600 letters from approximately 100 s of a subject’s speech. Similarly, it is calculated that it takes 3–5 min for the correct prediction rate to reach its peak. Therefore, our machine learning model is fully applicable to real-world clinical practice.

The domains of cognitive function affected by dementia are heterogeneous, and it seems difficult to capture the various cognitive declines only by linguistic aspects. However, neurodegenerative diseases are thought to damage the neurons that control cognition, speech, and language processes, and consequently affect the linguistic aspects of patients^22^. If we consider that various cognitive functions lead to language dysfunctions, then it is also possible that various cognitive impairments are reflected in language. In fact, linguistic changes in AD patients are known to occur early in the course of the disease^23–27^, and language function has been found to be correlated with overall cognitive function^28^. In addition, previous studies using NLP and machine learning have shown that AD can be identified with high accuracy^10–18^.

While recent studies using NLP and machine learning to detect dementia have used tasks such as the picture description task to encourage spontaneous speech, the results of this study show that it is possible to screen for dementia using unstructured free conversation without requiring a specific task. A screening method using free conversation is simple and easy to disseminate as a screening method because it can be performed without a need for specific tools. Furthermore, telemedicine is now widely used in many countries, especially with the COVID-19 outbreak^29^, but cognitive function tests can be difficult to perform using telemedicine; instead, free-conversation screening might be useful as a remote screening tool. In addition, since free conversation does not have a correct answer, unlike task-based testing, learning effects might be less likely to occur.

The present study had the following limitations. In this study, MCI and CHC samples were combined into a non-dementia class, and binary classification versus dementia was performed. We plan to further investigate whether it is possible to discriminate between CHC and MCI, and perform a ternary classification as we accumulate more data. Moreover as with the MMSE and other screening methods, the education level and age of the subject may affect the results of this method, and this possibility will require further investigation. In addition, the interview used in this study was designed to encourage speech by asking simple questions such as “How are you feeling today?”, but since communication involves interactions, the score may be affected by the ability and attitude of the interviewer.

Although the number of datasets must be increased before application in actual clinical practice, the results of this study show that it is possible to construct a machine learning algorithm that can discriminate dementia from non-dementia with a high accuracy based on a free conversation without the use of a task. In the future, this methodology could be useful as a screening tool for dementia.

## Methods

### Data source

Data from the Project for Objective Measures using Computational Psychiatry Technology (PROMPT)^30^ was used in this study. This study was a prospective observational multicenter study performed in Japan with the goal of identifying objective markers using voice and speech, body motion, facial expression, and daily activity data for mood disorders and dementia. Participants were recruited at the psychiatry departments of 10 different medical facilities, and each ethics committee, including that of the Keio University School of Medicine, approved the study. All the participants provided written informed consent before participating in this study, which was designed in accordance with ethical principles based on the Declaration of Helsinki. The recruitment period was from March 9, 2016, to March 31, 2019. This study used data from patients diagnosed as having major neurocognitive disorder or mild neurocognitive disorder according to the Diagnostic and Statistical Manual of Mental Disorders 5 (DSM-5) criteria and from participants recruited as cognitively healthy controls (CHC). CHC were also screened for a history of mental disorders using the Mini-International Neuropsychiatric Interview (M.I.N.I.), and CHC with any history of psychiatric disorder were excluded. Individuals with apparent speech problems including aphasia and dysarthria were also excluded.

The participants were given 10 min to have an unstructured conversation with a psychiatrist or psychologist, such as an interview on the topic of their mood or daily living. During that time, their speeches were recorded with a microphone. After the interview, the Clinical Dementia Rating (CDR), Mini-Mental State Examination (MMSE), Logical memory of the Wechsler Memory Scale-Revised, and Geriatric Depression Scale (GDS) were evaluated. If the participant agreed, a similar interview was conducted and the above data were collected once again after a minimum interval of 4 weeks.

### Data eligibility

In the present study, we analyzed the data described above. To eliminate the effect of depressive symptoms on cognitive function, the resulting data was excluded from the analysis if a participant’s GDS was 10 or higher. In addition, data from subjects under the age of 45 years, data with missing conversational data or rating data, and data in cases where the subject spoke with a strong dialect were excluded from the analysis.

### Data labeling

In this study, we aimed to develop a system capable of screening for dementia. Therefore, we attempted to construct a machine learning model to discriminate between dementia and non-dementia, including CHC and MCI. Dementia and non-dementia were determined by three neuropsychological tests, namely the CDR, MMSE, and logical memory II. The cutoff for the logical memory II test was based on the education history: subjects with 0–9 years of education scored 2 points or less, subjects with 10–15 years of education scored 4 points or less, and subjects with 16 or more years of education scored 8 points or less. Dementia was defined as (1) CDR ≥ 1 and MMSE ≤ 23, (2) CDR ≥ 1, MMSE ≥ 24, and below the logical memory II cutoff, or (3) CDR = 0.5, MMSE ≤ 23, and below the logical memory II cutoff. Non-dementia (including MCI) was defined as CDR ≤ 0.5 and MMSE ≥ 24. If the patient showed patterns other than these categories, we labeled them as dementia or non-dementia based on their clinical diagnosis. The clinical labeling procedure based on the results of the neuropsychological tests is shown in Supplementary table.

To improve the accuracy of the machine learning, we decided to use data that reflects typical symptoms for training. Therefore, data that fell into the following categories were used not only as test data, but also as training data: dementia with CDR ≥ 1, MMSE ≤ 23, and logical memory II below the cutoff; non-dementia (MCI) with CDR = 0.5, MMSE ≥ 24, and logical memory II below the cutoff; and non-dementia (CHC) with CDR = 0, MMSE ≥ 24, and logical memory II above the cutoff. Data that did not meet these criteria were used only as test data.

In this study, data were acquired multiple times from the same participant, so it was possible for the same participant to have different states depending on the time when the conversational data was acquired (e.g., after conversion from MCI to dementia). Therefore, each data was labeled using the result of the cognitive evaluation performed at the time of the recording of the conversational data.

### Document embedding

From the recorded data, only the subject's speech was transcribed into text data, including fillers, and compiled into a single document. This document was transformed into a vector represented by 150-dimensional features using previously reported technology^31^. In the present study, we set the negative sampling value to 5 and the number of dimensions to 150, and we finally obtained a 150-dimensional document vector from the morpheme elements. In addition, the same method was used to create a 50-dimensional vector using bi-grams of parts of speech as input features, for a total of 200 dimensions for morphemes and parts of speech.

### Machine learning procedure

**Figure 4:**
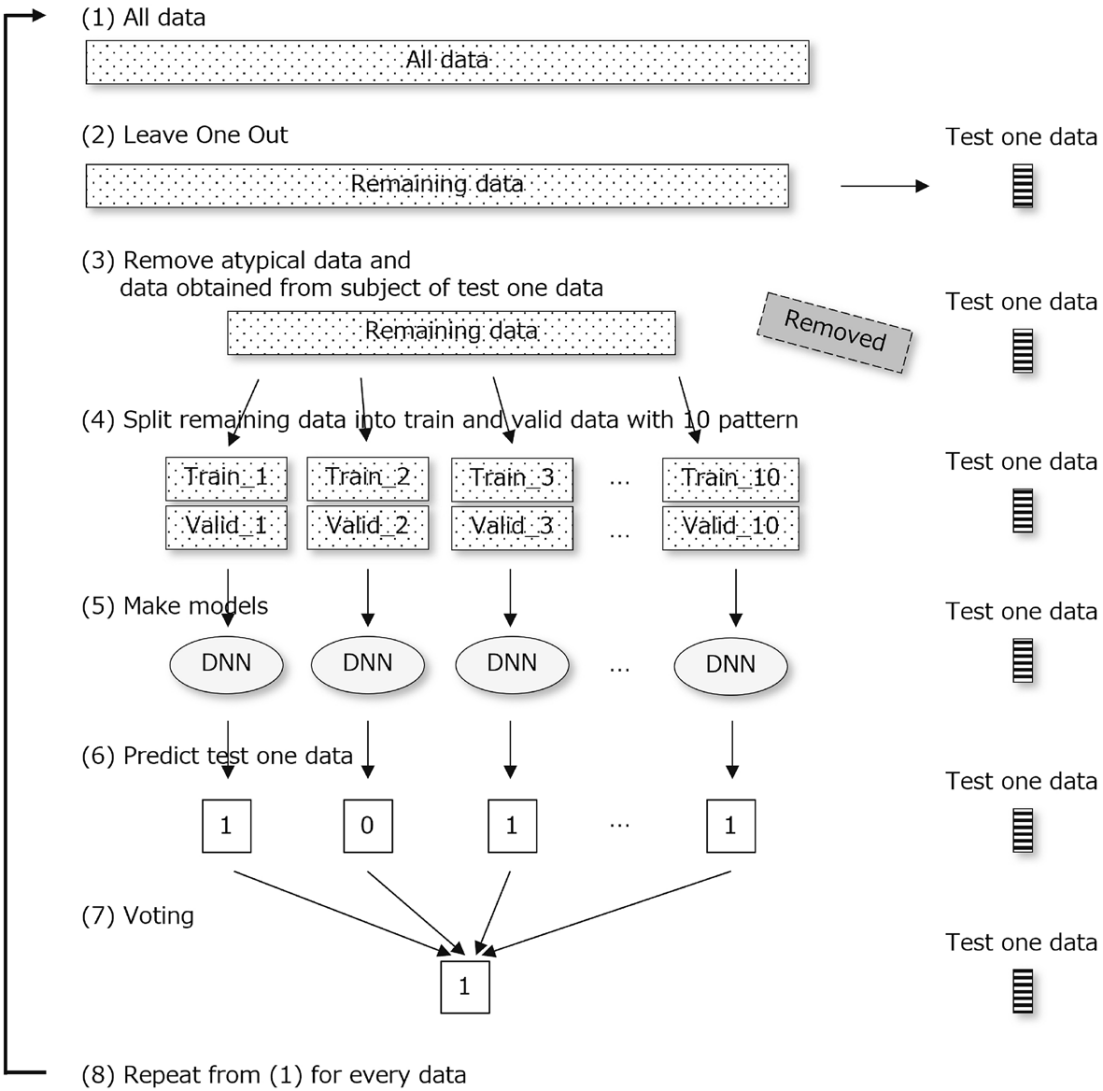
Architecture of the machine learning and validation methods.

In this study, we built a DNN-based prediction model that discriminated between two classes of dementia and non-dementia. The DNN model was constructed using Python 3.6, tensorflow 2.20 library, and a five-layer neural network consisting of an input layer, three hidden layers, and an output layer. The various hyperparameters were optimized using Optuna 2.0.0. Leave-One-Out Cross-Validation (LOOCV) was used for model building and performance evaluation. Since it was possible for multiple data acquisitions to be obtained from the same subject in this study, there was a risk that speech data from the same subject could be used in both the validation and training sets, which would improve the apparent accuracy. To avoid this effect, we added a process to exclude text data from subjects who had provided validation data from being used as training data. The details are as follows. The architecture of the machine learning and validation methods is depicted in the Fig. 4.

(i) Extract one test data from all data.
(ii) From the remaining data, exclude data from the same subjects as the test data and data that do not meet the criteria for training.
(iii) Randomly split the remaining data so that the ratio of dementia to non-dementia is kept constant and the ratio of training data to validation data is 3:1. To consider the effect of random splitting, create 10 sets of training and validation datasets with different splits.
(iv) Build 10 prediction models with the 10 sets of training and validation datasets.
(v) Repeat the above steps from i to iv for the number of samples.

The prediction accuracy of the results of voting by the 10 prediction models was calculated for one test data. The threshold for the number of votes that determines the prediction for the entire model was adopted so as to yield the highest accuracy. Thereafter, the accuracy, sensitivity, and specificity in this setting were used as evaluation indices for the prediction models. For the purpose of calculating AUC, Receiver Operating Characteristic (ROC) curves were created for the 10 models used for voting, and the average AUC was calculated.

As a sub-analysis, we also evaluated the prediction accuracy when the data were divided into two groups according to sex and according to age (75 years and older vs. less than 75 years).

### Relationship between the number of letters and prediction accuracy

To examine the effect of utterance length on the prediction accuracy, we prepared text data with different document lengths in units of 100 letters from the beginning of each text and converted each of them into a 200-dimensional vector. Predictions were made on this vector using a model built with LOOCV, and the document length and prediction accuracy were evaluated. To predict each vector, we used a model designed to predict the original document before changing the document length.

### Verification of vectorization and machine learning algorithms

To compare our document embedding as well as machine learning procedure with other methods, we calculated the prediction accuracy using TF-IDF and BERT for the vectorization and using Naive Bayes, Logistic regression, Support Vector machine, and XGBoost for machine learning, respectively. In the XGboost model, 10 sets of training and validation patterns were created for one test data extracted by LOOCV, and prediction by voting using 10 models was performed. We also performed vectorization using TF-IDF and Japanese BERT, and calculated the prediction accuracy of voting using the 10 models trained by DNN.

## Supplementary Information

Supplementary Information.

## Data Availability

The datasets used in this study are not publicly available.
