# Supplementary material for: Identifying neurocognitive disorder using vector representation of free conversation
Source: Sci Rep. 2022 Aug 3;12:12461. doi: 10.1038/s41598-022-16204-4 (PMC9349220; doi:10.1038/s41598-022-16204-4)
Supplement: Supplementary file 1 — Supplementary Information. [file 41598_2022_16204_MOESM1_ESM.pdf]

CDR	MMSE	LM II	Labels
0	≧24	More than cutoff	Non-dementia (CHC that meets the criteria for training)
0	≧24	Less than cutoff	Non-dementia (CHC or MCI)
0	≦23	More than cutoff	Classified by medical record survey
0	≦23	Less than cutoff	Classified by medical record survey
0.5	≧24	More than cutoff	Non-dementia (CHC or MCI)
0.5	≧24	Less than cutoff	Non-dementia (MCI that meets the criteria for training)
0.5	≦23	More than cutoff	Classified by medical record survey
0.5	≦23	Less than cutoff	Dementia
≧1	≧24	More than cutoff	Classified by medical record survey
≧1	≧24	Less than cutoff	Dementia
≧1	≦23	More than cutoff	Dementia
≧1	≦23	Less than cutoff	Dementia (meets the criteria for training)

CDR = Clinical Dementia Rating. MMSE = Mini-Mental State Examination. LM II = logical memory delayed recall of Wechsler Memory Scale-Revised. Non-dementia include cognitively healthy controls and participants with mild cognitive impairment.

Supplementary Table: Relationship between neuropsychological test results and labels
